# Análisis situacional de los profesionales de enfermería para el desarrollo de un plan estratégico en un hospital de alta complejidad

**DOI:** 10.23938/ASSN.1116

**Published:** 2025-10-28

**Authors:** Beatriz Elena Martín Rivera, Laura Clavero López, María Gudiño Vázquez, Alicia Albalat Rodríguez, Marina de la Matta Cantó, Ana Castillo Ayala

**Affiliations:** 1 Hospital Universitario Ramón y Cajal Fuencarral-El Pardo Madrid España; 2 Área Funcional de Calidad, Docencia e Investigación Hospital Universitario Ramón y Cajal Fuencarral-El Pardo Madrid España; 3 Unidad de Calidad de Enfermería Hospital Universitario Ramón y Cajal Fuencarral-El Pardo Madrid España; 4 Unidad de Investigación en Cuidados Hospital Universitario Ramón y Cajal Fuencarral-El Pardo Madrid España

**Keywords:** Satisfacción Laboral, Agotamiento Profesional, Personal de Enfermería, Compromiso Laboral, Atención de Enfermería, Job Satisfaction, Burnout, Professional, Nursing Staff, Work Engagement, Nursing Care

## Abstract

**Fundamento:**

Analizar situación emocional, motivación y satisfacción del personal de enfermería para diseñar un plan estratégico de prevención del desgaste emocional.

**Metodología:**

Estudio transversal multietápico, desarrollado entre diciembre 2021 y febrero 2023 en un hospital terciario de alta complejidad, con profesionales dependientes de la Dirección de Enfermería (enfermería, fisioterapia, terapia ocupacional, personal técnico en cuidados auxiliares de enfermería, personal técnico auxiliar de farmacia) con experiencia laboral ≥2 años y antigüedad ≥2 meses en la unidad asignada. Se recogieron 65 ítems: variables sociodemográficas, reelección de la profesión, análisis emocional (cuestionario EVEA), satisfacción laboral (cuestionario Font-Roja) y nivel de conocimientos sobre factores internos del hospital (cuestionario autodiseñado).

**Resultados:**

Se incluyeron 596 profesionales. El 56% llevaba ≤10 años en el hospital. El 59% reelegirían la profesión. Se obtuvieron altos niveles de ira, ansiedad y tristeza, excepto en fisioterapeutas, y el 78% presentaba ≥7 puntos en cansancio y dificultad para desconectar. El 70% destacó buena comunicación con el supervisor, pero detectando limitaciones en su liderazgo. El turno o categoría profesional se asociaron con variables emocionales (ira, ansiedad) y de satisfacción. Se identificaron siete áreas de mejora para establecer una estrategia; las demandas más frecuentes se encuadraron en gestión de recursos humanos.

**Conclusiones:**

Los altos niveles de desgaste en enfermería, en comparación con fisioterapia, requieren estrategias específicas. Se necesita reforzar el liderazgo de los supervisores. Los resultados permitieron diseñar un programa de mejora alineando objetivos institucionales y necesidades percibidas por el equipo asistencial.

## Abstract (en)

**Background:**

This study aims to describe the emotional state, motivation, and satisfaction of nursing staff in order to design a management strategy to prevent burnout.

**Methodology:**

A three-phase, cross-sectional study was conducted between December 2021 and February 2023 in a high-complexity hospital. Participants included professionals under the Nursing Directorate (nursing, physiotherapy, occupational therapy, auxiliary nursing care technician, and pharmacy auxiliary technicians) with ≥2 years of employment and ≥2 months in their current department. Data were collected using 65 items covering sociodemographic variables, willingness to choose the same profession again, emotional state (EVEA questionnaire), job satisfaction (Font-Roja questionnaire), and knowledge of internal organizational factors (self-designed questionnaire).

**Results:**

A total of 596 professionals participated; 56% had ≤10 years of experience in the hospital and 59% would choose the same profession again. The EVEA questionnaire revealed high levels of anger, anxiety, and sadness. Font Roja scores indicated fatigue and difficulty disconnecting from work in 78% of participants. Although 70% responded good supervisor communication, limited leadership was identified. Associations were found between work shift, professional category, and emotional and satisfaction variables. Seven areas for improvement were identified to guide a strategic plan, with the most frequent demands related to human resources management.

**Conclusions:**

Nurses exhibits higher burnout levels than physiotherapists, emphasizing the need for targeted interventions. Strengthening supervisory leadership and implementing tailored management strategies can align institutional goals with the expressed need of care teams.

## INTRODUCCIÓN

El clima organizacional y la satisfacción laboral se consideran factores interrelacionados al considerarse determinantes del éxito organizacional, el desempeño profesional y el rendimiento laboral^1^^,^^2^.

El envejecimiento poblacional y el consiguiente aumento de las demandas sociales y de salud^3^, los avances tecnológicos o la transformación hacia la salud digital^4^ son algunos de los cambios que exigen la adquisición de nuevas competencias a los profesionales y una gestión estratégica y coordinada a las organizaciones, que les permita responder flexible y eficazmente a los desafíos emergentes, garantizando la calidad asistencial en un entorno cada vez más complejo^5^.

La enfermería ocupa un lugar destacado en el ámbito sanitario al constituir el grupo profesional más numeroso, y predominantemente femenino (353.635 profesionales siendo mujeres 297.778^6^), cuya labor asistencial influye directamente en indicadores clave como la morbi-mortalidad^7^^-^^9^, percepción de seguridad^10^ y la satisfacción de los pacientes, especialmente en relación con la calidad de los cuidados que reciben^11^^,^^12^.

La insatisfacción laboral depende tanto factores directos^13^, como indirectos^14^ que, mantenidos en el tiempo, afectan al estado de salud aumentando el absentismo^15^, los incidentes que afectan al paciente durante la práctica asistencial^10^^,^^16^ y la insatisfacción del personal enfermero, llevándoles a plantearse no continuar con la profesión o, incluso, abandonarla finalmente^17^. 

Parte de las acciones que han mostrado su efectividad pasan porque las instituciones dispongan de canales de comunicación fluidos^18^, desarrollen estrategias específicas de apoyo^19^ e incentiven mayor participación de los profesionales como forma de aumentar el sentido de pertenencia y compromiso^20^, logrando así aunar gestión sanitaria y práctica clínica orientadas como bien intrínseco y muestra de una ética empresarial que incluye a todos los estamentos involucrados. La crisis surgida con la pandemia, en la que España fue uno de los países más afectados durante la primera ola^21^, hizo emerger carencias hasta entonces sobrellevadas y el desarrollo de altísimos niveles de estrés y soledad, lo que hizo verse sobrepasados tanto instituciones como trabajadores, dejando una impronta significativa^22^^,^^23^. Esto refleja una realidad que debe abordarse sin dilación como herramienta fundamental ante nuevos acontecimientos.

Conocer a los profesionales vinculados a una institución, así como entender qué les afecta y de qué manera, se convierten en elementos fundamentales para el desarrollo de planes estratégicos que busquen convertirse en garantes de acciones sistematizadas a corto, medio y largo plazo que mejoren su satisfacción y su compromiso con la institución para alcanzar los estándares de excelencia deseados en la atención y cuidado del paciente.

Por ello, la Dirección de Enfermería de nuestro hospital, siguiendo las líneas trazadas en los planes de humanización y el Manual de Prevención del Desgaste Profesional del SERMAS^24^, se planteó un estudio cuyo objetivo fue analizar la situación emocional, el grado de motivación y la satisfacción laboral de su personal para desarrollar un programa estratégico de mejora orientado al cuidado y autocuidado del personal de enfermería con el propósito de prevenir el desgaste emocional y promover el bienestar profesional en el entorno hospitalario.

## METODOLOGÍA

Estudio multietápico mixto (descriptivo, analítico y de intervención) estructurado en fases sucesivas realizado en el Hospital Universitario Ramón y Cajal de diciembre de 2021 a febrero de 2023 mediante técnicas de consenso de grupos de expertos y encuestas a los equipos asistenciales dependientes de la Dirección de Enfermería.

Se incluyeron participantes en activo con, al menos, 2 años de actividad laboral en nuestro hospital. Se excluyó a quienes llevaban menos de dos meses en el área de destino o con funciones administrativas.

Se trabajó con un muestreo por cuotas atendiendo a la proporción de los estratos según área de trabajo y categoría profesional dentro de las mismas: personal de grado universitario (enfermería, fisioterapia [FT] y terapia ocupacional [TO]) y de grado medio de formación profesional (técnico auxiliar de farmacia [TAF] y técnico en cuidados auxiliares de enfermería [TCAE]) de las áreas de hospitalización, rehabilitación, laboratorios, cuidados críticos, urgencias, consultas, radiología, centros de especialidades, quirófanos y farmacia. Para el cálculo del tamaño de muestra se consideró p=q=0,5 con un nivel de confianza del 95%, una precisión del 20% y un 10% de reposiciones precisándose un total de 915 profesionales (514 grado universitario, 401 grado medio de formación profesional).

El estudio se desarrolló en tres fases.

### Fase 1. *Preparación y selección de herramientas* (diciembre 2021-abril 2022)

Esta primera fase incluyó la adecuación del plan con los objetivos institucionales mediante revisión de la literatura para identificar las variables críticas que inciden en el entorno laboral y en los niveles de satisfacción, completándola con reuniones técnicas *one to one* entre subdirecciones y Dirección de Enfermería con el equipo de supervisión de área funcional (SAF). Se identificaron las potenciales variables a analizar, se diseñó el estudio y se seleccionaron los instrumentos de medida atendiendo a criterios como validez, facilidad de lectura y número de ítems. Se elaboraron un mapa de impacto y un diagrama Gantt para la gestión y planificación del proyecto. 

Se desarrolló una sesión informativa para SAF y el equipo de supervisión de unidad (SU) explicando los fundamentos del estudio, su importancia y las acciones previstas que posteriormente transmitieron a sus equipos asistenciales.

### Fase 2. *Estudio descriptivo transversal* (abril-septiembre 2022)

A través de la plataforma Google, se diseñó un cuestionario de 65 ítems que integraba cuatro bloques de preguntas:

1. *Variables sociodemográficas*: cuatro ítems en formato multirrespuesta recogiendo categoría profesional (enfermería, fisioterapia-terapia ocupacional, TCAE, TAF), área de trabajo, años trabajados en el HURyC (<5, 5-10, 11-20, 21-30, >31) y turno actual (mañana, tarde, rotatorio). Se añadió la pregunta de si volverían a elegir su profesión (sí/no). No se incluyó la variable sexo a fin de evitar la generación de medidas que pudieran resultar excluyentes, o reforzar estereotipos de género. Se ha asignado género femenino al personal de enfermería y TCAE, dado el contexto de predomino femenino, pero incluye tanto mujeres como hombres.
2. *Estado emocional*: mediante el cuestionario EVEA validado por Jesús Sanz en 2001^25^, analiza cuatro estados emocionales (depresión, ansiedad, hostilidad y alegría) a través de 16 ítems -cuatro para cada estado- con respuesta tipo Likert de 0 a 10. La consistencia interna (α de Cronbach) media y su rango para las distintas subescalas es: tristeza-depresión α=0,88 (0,86-0,92), ansiedad: α=0,92 (0,92-0,94), ira-hostilidad: α=0,93 (0,93-0,95), y alegría: α=0,92 (0,88-0,96).
3. *Nivel de satisfacción y motivación*: mediante el cuestionario Font Roja validado en 1998 por Jesús María Aranaz^26^, consta de 24 ítems con respuesta tipo Likert de 5 puntos agrupados en nueve factores (satisfacción con el trabajo, tensión relacionada con el trabajo, competencia profesional, tensión en el trabajo, grado en el que un individuo cree que puede mejorar, relación interpersonal con sus jefes, relación interpersonal con compañeros, características extrínsecas del estatus, y monotonía laboral). El resultado se denomina satisfacción media global (SMG) que oscila entre -2 y 2 puntos y se categoriza en satisfechos (>0), indiferentes o neutros (0) y no satisfechos (<0). El α de Cronbach es >0,80 para todos los ítems excepto para el número catorce, cuyo valor de correlación ítem-total corregido fue <0,40.
4. *Nivel de conocimiento sobre información relativa a factores internos de nuestro hospital* (objetivos y liderazgo, área/unidad donde se desarrolla la actividad, y servicios al trabajador) mediante veinte ítems (trece en formato dicotómico, uno en formato multirrespuesta, cinco como pregunta abierta condicionada a la respuesta previa y una pregunta abierta sin condición).

El cuestionario no exigía cumplimentar todos los ítems. No se recopilaron datos personales ni direcciones de correo electrónico de los participantes para mantener el anonimato. Se distribuyó mediante un código QR enviado vía e-mail y difundido mediante carteles en los cuartos de estar. Se repitió el envío quincenalmente hasta completar el periodo de recogida (tres meses). Las supervisoras de unidad reforzaron y apoyaron el seguimiento, así como la resolución de dudas en sus unidades.

Se cumplió con los principios éticos establecidos en la Declaración de Helsinki y se consultó al Comité de Ética de la Investigación. Se pidió consentimiento a todas las personas participantes y se les informó sobre sus derechos y protección de datos según legislación vigente. Se aseguró la confidencialidad de los datos mediante sistemas de anonimización.

### Fase 3. *Diseño del plan estratégico* (octubre 2022-febrero 2023)

Según los resultados de fase 2 se diseñó el plan estratégico de mejora, estableciendo objetivos a corto, medio y largo plazo centrados en las áreas demandadas, con cambios factibles en procesos clave para la organización. Para su implementación, en una nueva reunión con el equipo de gestión se difundieron los resultados y la estrategia a seguir. Se elaboró un nuevo cronograma y una matriz RACI (Responsable-Aprobador-Consultado-Informado). Se planteó una evaluación en diciembre de 2024 para verificar y dimensionar el impacto de las intervenciones.

### Análisis estadístico

Las variables recogidas en la fase 2 del estudio se describieron como frecuencias y porcentajes si eran cualitativas, y se compararon con χ^2^. Las variables cuantitativas se describieron con la media y la desviación estándar (DE); atendiendo a la normalidad de su distribución, las variables se compararon con t de Student, Anova, o Pearson -según las variables analizadas-, o sus correlativos no paramétricos. Para el análisis estadístico se utilizó el programa SPSS v26. Se consideraron significativos los valores bilaterales de p <0,05.

## RESULTADOS

De los 915 cuestionarios enviados, se recibieron 608 (66,4%). Se anularon 12 por su bajo índice de respuesta en los cuestionarios EVEA o Font Roja, conformando la muestra final 596 participantes (65%).

El 63% de los profesionales participantes fueron enfermeras, el 57% llevaba trabajando 10 años o menos en nuestro hospital y el 58% tenían turno rotatorio. El mayor número de respuestas provinieron del área de hospitalización (28%) y de críticos (20%) (Tabla 1).

**Tabla 1:** Características del personal participante en el estudio

Variables	n (%)	
*Categoría profesional*		
ENF	373	(63)
FT/TO	31	(5)
TCAE	178	(30)
TAF	14	(2)
*Área de trabajo*		
Hospitalización	166	(28)
Urgencias	96	(16)
Quirófano	71	(12)
Críticos	121	(20)
Rehabilitación	30	(5)
Centros Salud Mental	6	(1)
Consultas	83	(14)
Laboratorios	7	(1)
Radiología	16	(3)
*Turno*		
Mañana	182	(31)
Tarde	67	(11)
Rotatorio	347	(58)
*Antigüedad en el hospital*		
<5 años	212	(36)
5-10 años	126	(21)
11-20 años	171	(29)
21-30 años	55	(9)
>30 años	32	(5)

ENF: enfermería; FT: fisioterapia; TO: terapia ocupacional; TCAE: técnico en cuidados auxiliares de enfermería; TAF: técnico auxiliar de farmacia.

El 100% de participantes respondió a la pregunta sobre si reelegiría su profesión, y el 59% respondieron afirmativamente. Reelegirían su profesión el 100% de quienes trabajan en centros de Salud Mental, el 93% de TAF y el 71% de quienes trabajaban de mañana, mientras que el personal del área de urgencias fue quien menos la reelegiría (44,8%) (Tabla 2).

**Tabla 2:** Reelección de su profesión según variables sociodemográficas

Variables	n (%)	p
*Categoría*		
ENF	203 (54,4)	<0,001
FT/TO	22 (71)	
TCAE	115 (64,6)	
TAF	13 (92,9)	
*Área de trabajo*		
Hospitalización	95 (57,2)	<0,001
Urgencias	43 (44,8)	
Quirófano	41 (57,7)	
Críticos	69 (57)	
Rehabilitación	22 (73,3)	
Centros Salud Mental	6 (100)	
Consultas	63 (75,9)	
Laboratorios	5 (71,4)	
Radiología	9 (56,3)	
*Turno*		
Mañana	130 (71,4)	0,244
Tarde	38 (56,7)	
Rotatorio	185 (53,3)	
*Antigüedad en el hospital*		
<5 años	212 (35,6)	0,880
5-10 años	126 (21,1)	
11-20 años	171 (28,7)	
21-30 años	55 (9,2)	
>30 años	32 (5,4)	

ENF: enfermería; FT: fisioterapia; TO: terapia ocupacional; TCAE: técnico en cuidados auxiliares de enfermería; TAF: técnico auxiliar de farmacia.

Todas las preguntas del cuestionario EVEA fueron respondidas por el ≥99% de participantes. El 58% presentó ≥7 puntos en la subescala ansiedad, 61% en ira-hostilidad, 34% en tristeza-depresión y 15% en alegría. Por categorías, el personal TAF mostró los mayores niveles de ansiedad (78,5%) frente a FT/TO que presentaron los más bajos (22,5%), lo que se repitió con la subescala ira-hostilidad (71,2 vs 12,8%) y tristeza-depresión (57 vs 6,4%). En cuanto a la subescala alegría, FT/TO presentó los valores más altos frente a enfermería, quienes tenían los menores niveles de alegría (67,8 vs 11%).

El cuestionario Font Roja también fue cumplimentado por el ≥99% en todas las preguntas. Llamó la atención que el menor índice de respuesta se presentó en F7 (Relación interpersonal con compañeros, 590 respuestas). Destacaron cuestiones como alto nivel de cansancio al acabar la jornada (78%), incapacidad de desconectar totalmente al salir del hospital (78%) y afectación del estado de ánimo y de las horas de sueño (75% cada una de ellas). En contraposición, las personas participantes presentaron mucho interés por las actividades realizadas (71%) y se veían bien capacitadas para desarrollar su trabajo (73%). La SMG general fue de 0,00 (0,32) lo que muestra un resultado neutro. La dimensión positiva más valorada fue la relación con los compañeros (3,8; DE=1,2), independientemente de la categoría profesional, mientras que entre las negativas fue la presión por el trabajo (3,7; DE=1,2) (Tabla 3). No se encontraron relaciones estadísticamente significativas respecto al tiempo trabajado y los factores del cuestionario EVEA, pero sí respecto a la SMG (p=0,005). El análisis *post-hoc* mostró diferencias significativas entre el personal de FT/TO y el resto de profesionales, con menores niveles de ansiedad, ira y depresión, y mayor nivel de alegría; la SMG fue ligeramente positiva mientras que en el resto fue ligeramente negativa, pero sin alcanzar significación estadística.

**Tabla 3:** Puntuaciones obtenidas en los cuestionarios EVEA y Font Roja según variables sociodemográficas

	EVEA				Font-Roja
	Media (DE)				Media (DE)
	Ansiedad	Ira	Depresión	Alegría	SMG
*Categoría*					
ENF	6,93 (2,19)	7,25 (2,37)	6,02 (2,29)	4,56 (1,90)	-0,11 (0,67)
FT/TO	4,19 (2,54)	3,44 (2,56)	3,52 (2,17)	7,27 (1,76)	0,48 (0,63)
TCAE	6,94 (2,48)	7,17 (2,65)	6,30 (2,54)	4,78 (2,28)	-0,14 (0,69)
TAF	7,47 (2,41)	7,33 (2,82)	7,12 (2,14)	4,54 (1,84)	-0,24 (0,48)
*Valor p*	0,024	0,002	<0,001	<0,001	0,074
*Área*					
Hospitalización	7,23 (2,08)	7,58 (2,27)	6,45 (2,15)	4,24 (1,93)	-0,19 (0,62)
Urgencias	7,77 (2,04)	8,27 (1,88)	7,23 (2,10)	3,85 (1,84)	-0,42 (0,69)
Quirófanos	6,87 (2,07)	7,43 (2,05)	5,8 (2,19)1	4,82 (1,80)	0,01 (0,64)
Críticos	6,68 (2,34)	7,17 (2,39)	5,94 (2,20)	4,87 (1,95)	0,00 (0,65)
Rehabilitación	4,37 (2,44)	3,44 (2,18)	3,58 (2,17)	6,90 (2,02)	0,44 (0,73)
CSM	3,96 (2,38)	3,88 (3,31)	3,01 (2,16)	5,78 (1,88)	0,56 (0,30)
Consultas	5,98 (2,77)	5,50 (2,98)	4,90 (2,76)	5,86 (2,21)	0,05 (0,71
Laboratorios	7,07 (1,74)	6,27 (2,19)	6,17 (2,69)	4,58 (2,02)	-0,03 (0,55)
Radiología	7,10 (1,89)	7,27 (2,24)	6,38 (2,09)	4,74 (2,02)	-0,01 (0,53)
*Valor p*	0,004	<0,001	<0,001	<0,001	<0,001
*Antigüedad en el hospital*					
<5 años	6,82 (2,39)	6,97 (2,58)	6,01 (2,30)	5,10 (1,97)	-0,02 (0,70)
5-10 años	6,98 (2,27)	7,18 (2,53)	6,20 (2,53)	4,55 (2,17)	-0,15 (0,66)
11-20 años	6,83 (2,29)	7,13 (2,58)	6,00 (2,34)	4,64 (2,07)	-0,06 (0,66)
21-30 años	6,63 (2,69)	6,91 (2,75)	5,96 (2,67)	4,35 (2,29)	-0,22 (0,74)
>31 años	6,23 (2,71)	6,52 (3,03)	5,11 (2,75)	4,79 (2,18)	-0,20 (0,70)
*Valor p*	0,901	0,596	0,236	0,12	0,005
*Turno*					
Mañana	5,73 (2,63)	5,49 (2,90)	4,88 (2,54)	5,81 (2,16)	0,10 (0,74)
Tarde	6,99 (2,24)	7,26 (2,51)	6,18 (2,45)	4,78 (2,03)	0,05 (0,68)
Rotatorio	7,33 (2,07)	7,79 (2,06)	6,55 (2,15)	4,22 (1,85)	-0,22 (0,63)
*Valor p*	0,513	0,709	0,008	0,533	0,576
*Total*	6,81 (2,38)	7,03 (2,61)	6,00 (2,43)	4,77 (2,09)	-0,09 (0,68)

DE: desviación estándar; SMG: satisfacción media global; ENF: enfermería; FT: fisioterapia; TO: terapia ocupacional; TCAE: técnico en cuidados auxiliares de enfermería; TAF: técnico auxiliar de farmacia; CSM: centros de salud mental.

Respecto al cuestionario autodiseñado, casi todas las preguntas fueron respondidas por el ≥99% de participantes, salvo P3 (*Considera que su supervisor de unidad tiene actitudes y habilidades necesarias para ser buen líder*) y P5 (*Se siente respaldado por los mandos superiores*), respondidas por el 97% respectivamente.

Destacó la categoría de TAF por presentar el mayor número de respuestas negativas (en casi todas las preguntas).

El aspecto más conocido fueron las actividades a desempeñar en su unidad según competencias (P10: 92,4%), que mostró diferencias por área: 93% en todas las categorías salvo 77% en TAF, seguido de conocer quién es su SAF (P2: 85,2%), donde FT/TO presentaron el mayor porcentaje (87,1%) y TAF el menor (78,6%), y conocer los protocolos de su unidad (P9: 77%) donde solo el 33% de TAF los conocían. Destaca la buena comunicación con la persona supervisora de unidad (P4: 70%) motivada, principalmente, por su accesibilidad; sin embargo, un 40,4% no las consideraban buenos líderes (P3) debido a falta de empatía o ser de reciente nombramiento, destacando la diferencia entre TAF (69,2%) y FT/TO (3,2%).

Dado que desde octubre de 2019 se añadió un nuevo programa de gestión de la formación, se preguntó sobre los canales de información de esta para evaluar su efectividad. Las vías principales fueron el correo electrónico (38%) y la *app* (43%) (Tabla 4).

**Tabla 4:** Frecuencia de respuestas afirmativas (formato dicotómico) sobre el nivel de conocimientos de la institución y relaciones laborales con el equipo de gestión según categoría, área de destino, turno de trabajo y antigüedad dentro el hospital

	Respuesta afirmativa											
	n (%)											
	P1	P2	P3	P4	P5	P6	P7	P8	P9	P10	P11	P12
Categoría profesional												
ENF	207 (55)	316 (84.7)	218 (59,6)	257 (69,3)	88 (240,)	270 (72,6)	235 (62,2)	203 (54,7)	294 (79,2)	343 (92,7)	143 (38,5)	166 (45,1)
FT/TO	15 (48,4)	27 (87,1)	30 (96,8)	29 (93,5)	23 (74,2)	20 (64,5)	16 (51,6)	4 (12,9)	27 (87,1)	29 (93,5)	14 (45,2)	24 (77,4)
TCAE	96 (54,2)	153 (84,4)	94 (55,0)	122 (70,1)	50 (29,4)	124 (70,1)	109 (62,6)	63 (36,0)	133 (75,1)	163 (92,6)	61 (34,5)	71 (39,9)
TAF	7 (53,8)	11 (78,6)	4 (30,8)	6 (42,9)	3 (25,0)	5 (35,7)	7 (50,0)	1 (7,7)	4 (33,3)	10 (76,9)	1 (7,7)	3 (23,1)
*valor de p*	0,05	0,05	<0,001	<0,001	<0,001	0,02	0,47	<0,001	<0,001	0,2	0,08	<0,001
*Área*												
Hospitalización	92 (55,4)	124 (74,4)	84 (51,9)	115 (71,0)	17 (10,4)	112 (68,3)	116 (70,3)	94 (57,3)	133 (80,6)	158 (96,9)	59 (36)	80 (49,1)
Urgencias	48 (50,0)	85 (89,5)	34 (37,0)	45 (47,9)	21 (22,1)	73 (76,0)	40 (42,1)	25 (26,3)	61 (64,2)	77 (81,1)	6 (6,3)	28 (29,5)
Quirófano	42 (59,2)	66 (93,0)	42 (60,9)	45 (63,4)	28 (39,4)	52 (73,2)	39 (56,5)	26 (37,1)	56 (78,9)	62 (87,3)	29 (40,8)	22 (31,0)
Críticos	68 (56,7)	107 (88,4)	99 (81,8)	107 (88,4)	36 (31,0)	102 (84,3)	86 (71,1)	101 (83,5)	112 (93,3)	119 (98,3)	73 (60,3)	63 (52,1)
Rehabilitación	12 (40,0)	26 (86,7)	29 (96,7)	28 (93,3)	21 (72,4)	20 (66,7)	16 (53,3)	3 (10,0)	24 (80)	29 (96,7)	10 (33,3)	23 (76,7)
Centros SM	3 (50,0)	6 (100)	5 (83,3)	6 (100)	4 (66,7)	6 (100)	5 (83,3)	2 (33,3)	4 (66,7)	6 (100)	4 (66,7)	4 (66,7)
Consultas	46 (56,1)	74 (89,2)	46 (57,5)	56 (67,5)	32 (41,6)	42 (50,6)	54 (65,9)	16 (19,8)	55 (67,1)	76 (93,8)	29 (35,4)	32 (39,5)
Laboratorios	6 (85,7)	5 (71,4)	3 (42,9)	3 (42,9)	1 (14,3)	4 (57,1)	5 (71,4)	2 (28,6)	5 (71,4)	6 (85,7)	2 (28,6)	3 (42,9)
Radiología	8 (50,0)	14 (87,5)	4 (28,6)	9 (56,3)	4 (26,7)	8 (50,0)	6 (37,5)	2 (12,5)	8 (53,3)	12 (75,0)	7 (43,8)	9 (56,3)
*valor de p*	0,51	<0,001	<0,001	<0,001	<0,001	<0,001	<0,001	<0,001	<0,001	<0,001	<0,001	<0,001
*Turno*												
Mañana	113 (62,4)	163 (89,6)	122 (69,3)	140 (77,3)	88 (50,3)	123 (67,6)	115 (64,2)	58 (32,0)	139 (76,8)	168 (93,3)	73 (40,1)	94 (51,9)
Tarde	27 (40,9)	56 (83,6)	33 (51,6)	40 (60,6)	12 (19,0)	41 (62,2)	39 (58,2)	30 (45,5)	49 (75,4)	59 (89,4)	34 (51,5)	32 (49,2)
Rotatorio	185 (53,3)	288 (83,2)	191 (56,0)	234 (68,2)	64 (18,8)	255 (73,7)	213 (61,7)	183 (53,4)	270 (78,3)	318 (92,4)	112 (32,6)	138 (40,1)
*valor de p*	<0,001	0,13	<0,001	0,01	<0,001	0,09	0,67	<0,001	0,84	0,58	<0,001	0,26
*Antigüedad en el hospital (años)*												
<5	83 (39,5)	173 (81,6)	146 (70,2)	163 (77,3)	63 (30,6)	161 (75,9)	151 (71,2)	102 (48,3)	167 (79,9)	198 (94,3)	103 (49)	106 (50,7)
5-11	69 (54,8)	106 (84,1)	71 (57,7)	87 (69,0)	35 (28,2)	82 (65,1)	70 (56.0)	54 (43,2)	95 (75,4)	111 (88,8)	43 (34,4)	54 (43,2)
11-20	109 (63,7)	147 (86,5)	89 (54,3)	107 (64,1)	44 (26,5)	119 (70,4)	100 (59,9)	85 (50,3)	129 (76,3)	156 (92,3)	51 (30,0)	76 (44,4)
21-30	38 (69,1)	53 (96,4)	30 (55,6)	38 (70,4)	14 (26,4)	32 (58,2)	28 (50,9)	22 (40,7)	44 (80,0)	51 (92,7)	12 (21,8)	19 (35,8)
>30	26 (81,3)	28 (87,5)	10 (31,3)	19 (59,4)	8 (26,7)	25 (78,1)	18 (56,3)	8 (25,8)	23 (71,9)	29 (93,5)	10 (31,3)	9 (28,1)
*valor de p*	<0,001	0,08	<0,001	0,04	0,92	0,04	0,01	0.09	0,75	0,48	<0,001	0,07

ENF: enfermería; FT: fisioterapia; TO: terapia ocupacional; TCAE: técnico en cuidados auxiliares de enfermería; TAF: técnico auxiliar de farmacia.

P1: Conoce el organigrama de la Dirección de Enfermería; P2: Conoce a su supervisor de área funcional; P3: Considera que su supervisor de unidad tiene actitudes y habilidades necesarias para ser buen líder; P4: La comunicación con el SU es adecuada; P5: Se siente respaldado por los mandos superiores; P6: Tiene información actualizada de cambios/modificaciones en su unidad; P7: Conoce los objetivos específicos de la unidad; P8: Conoce si hay guía de acogida en su unidad; P9: Conoce los protocolos de su unidad; P10: Conoce las actividades a desempeñar en su unidad según competencias; P11: Cree que las instalaciones son adecuadas para el correcto desempeño de su profesión; P12: La formación ofertada es adecuada para mejorar sus competencias.

Finalmente se dejó una pregunta abierta para que los profesionales plantearan las cuestiones que considerasen que pudieran mejorar su nivel de satisfacción en cualquiera de sus ámbitos. El 57,2% de participantes (n=341) aportaron comentarios: El 59% se categorizaron como “recursos humanos” (falta de personal, exceso de cargas de trabajo, mejoras para la conciliación familiar, reactivar el *pool* de enfermería) y el 46% como “supervisión y gestión” (problemas con las planillas, mejora de la escucha y comunicación con el supervisor, supervisores con carencia de habilidades para la gestión). El 19 % se agruparon en la categoría “docencia” (falta de tiempo para realizar cursos dentro de la jornada laboral, asignación de menos cursos de los solicitados, necesidad de más información sobre este campo), el 18% en “aspectos psicológicos” (ansiedad, estrés, falta de valoración y motivación) y el 15% en “recursos materiales” (problemas con las instalaciones o recursos disponibles).

Las estrategias de mejora implantadas en la fase 3 tras el análisis realizado se categorizaron en siete áreas, cuyas acciones, actividades y tiempos se decidieron según su potencial impacto, la necesidad de recursos o de cambios estructurales, y si eran propuestas realistas y efectivas (Tabla 5).

Se diseñaron estrategias anuales, salvo en aquellas acciones que requerían procesos formativos o revisión de documentación extensa (PRT, PC y PNT) donde la estrategia se diseñó para cinco años.

**Tabla 5:** Áreas de mejora y acciones propuestas

Áreas	Acciones y actividades propuestas
Docencia	Aumentar la oferta formativa en distintos formatos (cursos, sesiones y píldoras)
	Formar en solicitud de acreditaciones
	Optimizar de los sistemas de difusión
	Facilitar la asistencia
Investigación	Fomentar la investigación interna
Calidad	Se estructuró un plan para revisar y actualizar estándares, protocolos (PRT), procedimientos (PC) y protocolos normalizados de trabajo (PNT), a través de:
	- creación de un grupo de trabajo especializado
	- diseño de un programa formativo para fortalecer una red colaborativa interna de expertos
Recursos humanos	Flexibilizar la jornada laboral teniendo en cuenta las particularidades de cada área
	Difundir planificaciones
	Dimensionar los equipos valorando cargas de trabajo
Recursos materiales	Identificar necesidades de nuevos recursos
	Análisis más efectivos de la calidad de los productos
	Mejorar la logística de distribución
Gestión	Mejorar la comunicación interna
	Trabajar con instrumentos estandarizados
	Desarrollar una matriz de correspondencia entre competencias de los profesionales y puestos de trabajo disponibles
	Sistematizar la evaluación del nivel de satisfacción
Aspectos psicológicos	Gestión del apoyo
	Facilitar recursos para desarrollar habilidades emocionales

## DISCUSIÓN

En este estudio analizamos los factores que influyen en la dinámica organizacional, abordando la satisfacción laboral, el estado emocional del equipo asistencial, y los procesos internos desde la perspectiva de los equipos asistenciales dependientes de la Dirección de Enfermería. Aunque los temas abordados no son novedosos, su enfoque multidimensional y multidisciplinar proporciona una compresión más profunda de las dinámicas laborales y organizacionales, aportando así una visión más rica y contextualizada, lo que permitió identificar áreas de tensión y oportunidades de mejora relevantes para la gestión organizacional.

La tasa de respuesta del 65% representa una muestra robusta. La repetición sistemática de la solicitud de cumplimentación, la facilidad de acceso al código QR y el constante apoyo por parte del personal supervisor, así como la posibilidad de cumplimentación en cualquier tipo de dispositivo, creemos fueron algunas de las estrategias clave para alcanzar esta tasa.

Los resultados en casi todas las categorías pusieron de manifiesto altos niveles de ansiedad, depresión, e ira, lo que podría ser un motivo para que los profesionales mostraran su intención de no reelegir su profesión (41%). La bibliografía recoge rangos muy amplios en intención de dejar la profesión (9% a 85%), fluctuando según países, niveles de satisfacción y compromiso organizacional^15^^,^^27^^,^^28^, por lo que cualquier resultado debe analizarse con cautela.

El estado de ánimo más positivo mostrado por FT/TO podría relacionarse con el estilo de liderazgo con el que se gestiona este equipo, ya que el tipo de liderazgo influye sobre los equipos^29^.

La sensación de sentirse poco respaldados por sus superiores, trabajar en un entorno que no consideran adecuado para el desarrollo de su actividad, o la percepción de excesiva carga de trabajo pueden mediar tanto en ese rechazo hacia su profesión como en factores influyentes en la satisfacción. El estudio de Escobar-Aguilar y col^10^ indicó que los equipos con bajos niveles de satisfacción y ambientes laborales tensionados tienden a disminuir la calidad y seguridad de la atención prestada.

Estudios realizados sobre la huella dejada por la pandemia muestran el sufrimiento de los profesionales y sus efectos^23^^,^^30^. Aunque nuestro estudio se realizó pasado un tiempo prudencial, cabe pensar que sus efectos tardarán en superarse y pueden influir en nuestros resultados. Esto no resta importancia a la necesidad de desarrollar habilidades que permitan reducir temores, ansiedad o sensación de culpa. 

La percepción de exceso de cargas de trabajo puede requerir optimizar las plantillas para controlar las horas trabajadas porque aunque, según una revisión Cochrane, el incremento de personal no está claramente relacionado con mejores resultados^31^, sí lo está el exceso de horas de trabajo^32^.

Los profesionales se valoraron competentes para desarrollar su labor pero indicaron que la formación continuada no era suficiente para continuar progresando en su nivel competencial. Esta necesidad puede responder a la evolución constante del entorno laboral y a la exigencia de una mayor especialización. El estudio RN4CAST^10^ o el de Griffiths y col^33^ mostraron la relevancia de la formación y de la adquisición de habilidades, ya que se asociaron a una atención de mayor calidad y una reducción en la mortalidad. Por ello, evaluar la percepción interna permite crear programas específicos adaptados a cada centro atendiendo a necesidades concretas.

Dado que la evidencia señala que trabajar el liderazgo y la comunicación intraorganizacional son aspectos fundamentales por relacionarse con mayores niveles de satisfacción^17^^,^^34^^,^^35^, es necesario trabajar con el personal supervisor y diseñar programas específicos para formarlo, dado que un alto porcentaje de participantes percibe carencias en las competencias de liderazgo de supervisores, a pesar de reconocer su accesibilidad y capacidad de escucha. Esto puede convertirse en una herramienta fundamental para diseñar estrategias que disminuyan los niveles de estrés tanto de profesionales como de supervisores.

El Hospital Universitario Ramón y Cajal está inmerso en un programa de actualización estructural para situarse en la vanguardia de los centros de alta complejidad, lo que podría justificar el elevado porcentaje de profesionales que considera que las instalaciones son adecuadas para el correcto desempeño de su profesión.

Como posibles limitaciones encontramos que, aunque el cuestionario EVEA remarca que el estado de ánimo debe referirse al momento actual, cabe la posibilidad de que algunos profesionales hayan reflejado un periodo más prolongado, por lo que los resultados mostrarían una posición menos neutra respecto al momento a evaluar.

Aunque se tomaron todas las medidas necesarias para que los resultados no se pudieran vincular con las personas informantes, es posible que algunas dudaran de dichas directrices al tratarse de un estudio promovido desde la Dirección, y que no respondieran con sinceridad. Dado que los cuestionarios trabajados mediante sistemas *online* tienen menor índice de respuesta que otros formatos, se intentó minimizar el impacto del formato elegido a través de recordatorios enviados desde la Dirección de Enfermería y su refuerzo por el equipo de SAF y SU.

Nuestros resultados deben interpretarse dentro de su contexto, entendiendo que las decisiones tomadas pueden no ser extrapolables a otros centros sanitarios, ya que existen particularidades propias en cada institución.

En conclusión, nuestro estudio reveló altos niveles de malestar emocional y bajos niveles de satisfacción laboral generalizados, lo que podría relacionarse con el pensamiento de no querer reelegir su profesión, principalmente entre enfermeras. También se evidencia la necesidad de reforzar el acompañamiento mediante formación y estrategias de apoyo emocional y psicológico. Se demandaron programas de formación continua orientados a la actualización de competencias técnicas y al fortalecimiento de habilidades transversales. Nuestra oferta se revisa anualmente pero, ante las limitaciones presupuestarias y con la opción de incorporar horas formativas a la jornada laboral, se necesitaría reforzar la corresponsabilidad del personal para alinearse con sus aspiraciones profesionales. Los resultados mostraron un limitado liderazgo de los supervisores. Esto requirió reflexionar sobre la necesidad de implementar programas específicos orientados al desarrollo de habilidades interpersonales, de gestión de equipos y resolución de conflictos. Que se perciban limitaciones entre vida personal y laboral, sobrecarga de trabajo o falta de personal justifica el desarrollo de estrategias que flexibilicen los horarios y turnos, además de medidas que favorezcan el equilibrio entre responsabilidades laborales y familiares.

Todo ello plantea la necesidad de implementar un plan estratégico de mejora organizacional, centrado en el desarrollo del talento humano, la optimización de los recursos humanos y la promoción de una cultura laboral saludable y sostenible. Está previsto realizar a partir de 2025 una evaluación sobre las estrategias de mejora implantadas, a fin de ver su efectividad. Tras este análisis, y con los resultados obtenidos, si es necesario se implantarán nuevas líneas de trabajo para mejorar los resultados.

## Data Availability

Se encuentran disponibles bajo petición al autor de correspondencia.
